# Supplementary material for: Polymorphic Characterization, Pharmacokinetics, and Anti-Inflammatory Activity of Ginsenoside Compound K Polymorphs
Source: Molecules. 2021 Apr 1;26(7):1983. doi: 10.3390/molecules26071983 (PMC8037814; doi:10.3390/molecules26071983)
Supplement: Supplementary file 1 [file molecules-26-01983-s001.pdf]

molecules

Article

Polymorphic Characterization, Pharmacokinetics, and Anti-Inflammatory Activity of Ginsenoside Compound K Polymorphs

Yun-Yan Kuang [1,†], Xuan Gao [2,†], Yi-Jun Niu [3], Xun-Long Shi [3] and Wei Zhou [1,*]

1 Department of Chemistry, Fudan University, 2005 Songhu Road, Shanghai 200438, China; yykuang@fudan.edu.cn
2 Children's Hospital, Fudan University, 399 Wanyuan Road, Shanghai 201102, China; naux1111@163.com
3 School of Pharmacy, Fudan University, 826 Zhangheng Road, Shanghai 201203, China; 20211030038@fudan.edu.cn (Y.-J.N.); xunlongshi@fudan.edu.cn (X.-L.S.)
* Correspondence: zhouw@fudan.edu.cn; Tel./Fax: +86-21-5423-7431
† These authors contributed equally to this work.

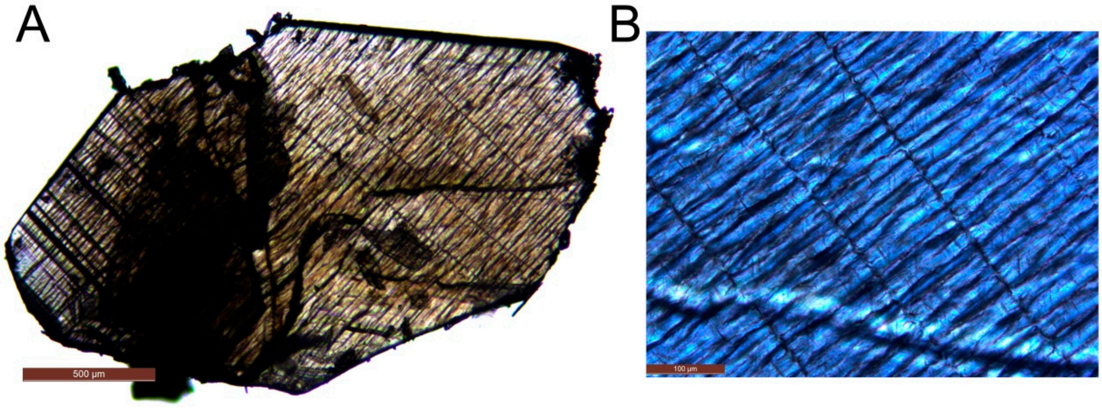

Figure S1. Optical microphotographs of Form IV by DMI 3000B optical inverted microscope (Leica, German): A) Magnification: 50×; B) Magnification: 200×.

Table S1. Plasma concentrations at different time points after intravenous injection of CK in rats (10 mg/kg).

Time (h)	Plasma concentrations of CK（ng/mL）			
	♂			
	1	2	3	Mean±SD
0.0083	78087.16	67670.83	69233.93	71663.98±5617.28
0.05	21702.04	11403.43	18155.87	17087.12±5231.83
0.1333	2609.85	1214.82	3002.82	2275.83±939.63
0.25	1261.15	664.52	1307.24	1077.63±358.51
0.5	393.16	214.44	391.89	333.16±102.82
1	103.46	75.42	107.64	95.51±17.52
2	60.30	41.16	54.90	52.12±9.87
4	42.63	32.27	85.89	53.60±28.44
6	19.70	14.03	27.30	20.34±6.66
9	9.16	11.73	16.36	12.42±3.64
Time (h)	♀			
	1	2	3	Mean±SD
0.0083	54838.90	127905.96	48362.17	77035.67±44173.82
0.05	15185.26	25786.82	6748.70	15906.92±9539.56
0.1333	1631.02	4937.02	980.77	2516.27±2121.49
0.25	672.83	1554.81	553.32	926.99±546.99

0.5	291.39	411.75	359.74	354.29±60.37
1	117.01	136.73	243.80	165.85±68.23
2	82.52	86.56	136.06	101.71±29.82
4	63.54	48.16	99.22	70.31±26.19
6	27.72	22.95	47.61	32.76±13.08
9	27.31	14.83	34.93	25.69±10.14

Table S2. Summary pharmacokinetic parameters after intravenous administration of CK to rats (10 mg/kg).

Parameters	♂			
	**1**	**2**	**3**	**Mean±SD**
$AUC_{(0-t)}$ [ug/L·h]	4264.50	2963.73	3980.16	3736.13±683.86
$AUC_{(0-\infty)}$ [ug/L·h]	4294.85	3012.98	4074.46	3794.10±685.38
$t_{1/2z}$ [h]	2.28	3.02	4.01	3.10±0.87
$MRT_{(0-t)}$ [h]	0.30	0.32	0.454	0.36±0.08
$MRT_{(0-\infty)}$ [h]	0.38	0.54	0.79	0.57±0.21
$V_{z/F}$ [L/kg]	15.34	28.92	28.37	24.21±7.69
$CL_{z/F}$ [L/h/kg]	4.66	6.64	4.91	5.40±1.08
**Parameters**	♀			
	**1**	**2**	**3**	**Mean±SD**
$AUC_{(0-t)}$ [ug/L·h]	3164.67	6150.84	2722.08	4012.53±1865.01
$AUC_{(0-\infty)}$ [ug/L·h]	3206.10	6173.20	2779.29	4052.86±1848.62
$t_{1/2z}$ [h]	2.44	1.95	2.14	2.18±0.25
$MRT_{(0-t)}$ [h]	0.57	0.26	1.00	0.61±0.37
$MRT_{(0-\infty)}$ [h]	0.94	0.34	1.44	0.91±0.55
$V_{z/F}$ [L/kg]	21.99	9.10	22.17	17.75±7.49
$CL_{z/F}$ [L/h/kg]	6.24	3.24	7.19	5.56±2.06

Note: $AUC_{(0-t)}$: area under the plasma concentration-time curve from zero to the time of the last quantifiable concentration; $AUC_{(0-\infty)}$: area under plasma concentration-time curve from zero to infinity; $t_{1/2z}$: half-life; $MRT_{(0-t)}$: mean residence time from time zero to the time for the last measurable concentration; $MRT_{(0-\infty)}$: mean residence time from time zero to infinity; $V_{z/F}$: apparent volume of distribution after intravascular administration; $CL_{z/F}$: the apparent plasma clearance of the drug after intravascular administration.

Table S3. Summary pharmacokinetic parameters after oral administration of Form I to rats (20 mg/kg).

Parameters	♂			
	**1**	**2**	**3**	**Mean±SD**
$AUC_{(0-t)}$ [ug/L·h]	762.48	1612.79	1074.22	1149.83±430.17
$AUC_{(0-\infty)}$ [ug/L·h]	762.82	1709.08	1092.09	1188.00±480.37
$t_{1/2z}$ [h]	2.06	2.87	3.98	2.97±0.96
T_{max} [h]	2.00	2.00	2.00	2.00±0.00
C_{max} [ug/L]	168.60	425.66	183.98	259.41±144.18
$V_{z/F}$ [L/kg]	77.79	48.49	105.00	77.09±28.26
$CL_{z/F}$ [L/h/kg]	26.22	11.70	18.31	18.74±7.27
**Parameters**	♀			
	**1**	**2**	**3**	**Mean±SD**
$AUC_{(0-t)}$ [ug/L·h]	2017.26	2120.78	2470.06	2202.70±237.26
$AUC_{(0-\infty)}$ [ug/L·h]	2050.82	2124.00	2473.25	2216.02±225.74
$t_{1/2z}$ [h]	4.03	2.57	2.56	3.05±0.84
T_{max} [h]	2.00	2.00	2.00	2.00±0.00
C_{max} [ug/L]	440.01	325.66	594.33	453.33±134.83
$V_{z/F}$ [L/kg]	56.64	34.89	29.88	40.47±14.22
$CL_{z/F}$ [L/h/kg]	9.75	9.42	8.09	9.09±0.88

Note: $AUC_{(0-t)}$: area under the plasma concentration-time curve from zero to the time of the last quantifiable concentration; $AUC_{(0-\infty)}$: area under plasma concentration-time curve from zero to in-

finity; $t_{1/2z}$: half-life; C_{max}: maximum concentration; T_{max}: time to maximum concentration; $V_{z/F}$: apparent volume of distribution after extravascular administration; $CL_{z/F}$: the apparent plasma clearance of the drug after extravascular administration.

Table S4. Summary pharmacokinetic parameters after oral administration of Form II to rats (20 mg/kg).

Parameters	♂			
	1	2	3	Mean±SD
$AUC_{(0-t)}$ [ug/L·h]	1095.34	774.36	1703.47	1191.06±471.90
$AUC_{(0-\infty)}$ [ug/L·h]	1198.51	780.14	1726.92	1235.19±474.45
$t_{1/2z}$ [h]	4.24	2.17	2.39	2.93±1.14
T_{max} [h]	2.00	2.00	2.00	2.00±0.00
C_{max} [ug/L]	187.99	197.22	311.79	232.33±68.97
$V_{z/F}$ [L/kg]	102.18	80.31	39.93	74.14±31.58
$CL_{z/F}$ [L/h/kg]	16.69	25.64	11.58	17.97±7.11
	♀			
	1	2	3	Mean±SD
$AUC_{(0-t)}$ [ug/L·h]	4557.73	1338.42	1445.59	2447.24±1828.52
$AUC_{(0-\infty)}$ [ug/L·h]	4823.11	1371.40	1459.17	2551.23±1967.99
$t_{1/2z}$ [h]	3.96	2.99	2.49	3.15±0.75
T_{max} [h]	2.00	2.00	2.00	2.00±0.00
C_{max} [ug/L]	777.00	209.65	392.12	459.59±289.63
$V_{z/F}$ [L/kg]	23.70	62.95	49.26	45.30±19.92
$CL_{z/F}$ [L/h/kg]	4.15	14.58	13.71	10.81±5.79

Table S5. Summary pharmacokinetic parameters after oral administration of Form III to rats (20 mg/kg).

Parameters	♂			
	1	2	3	Mean±SD
$AUC_{(0-t)}$ [ug/L·h]	656.21	1550.66	1308.63	1171.83±462.65
$AUC_{(0-\infty)}$ [ug/L·h]	662.23	1560.11	1328.10	1183.48±466.08
$t_{1/2z}$ [h]	3.40	2.68	3.83	3.30±0.58
T_{max} [h]	2.00	2.00	2.00	2.00±0.00
C_{max} [ug/L]	90.26	144.54	218.65	151.15±64.45
$V_{z/F}$ [L/kg]	148.10	49.55	83.24	93.63±50.09
$CL_{z/F}$ [L/h/kg]	30.20	12.82	15.06	19.36±9.46
	♀			
	1	2	3	Mean±SD
$AUC_{(0-t)}$ [ug/L·h]	1577.20	2580.19	1451.26	1869.55±618.65
$AUC_{(0-\infty)}$ [ug/L·h]	1582.68	2592.62	1513.29	1896.19±604.12
$t_{1/2z}$ [h]	2.82	3.06	2.56	2.81±0.25
T_{max} [h]	2.00	2.00	2.00	2.00±0.00
C_{max} [ug/L]	263.79	412.66	388.92	355.12±79.98
$V_{z/F}$ [L/kg]	51.35	34.03	48.90	44.76±9.37
$CL_{z/F}$ [L/h/kg]	12.64	7.71	13.22	11.19±3.02

Table S6. Summary pharmacokinetic parameters after oral administration of Form IV to rats (20 mg/kg).

Parameters	♂			
	1	2	3	Mean±SD
$AUC_{(0-t)}$ [ug/L·h]	540.26	711.78	1023.81	758.62±245.16
$AUC_{(0-\infty)}$ [ug/L·h]	603.16	720.70	1028.47	784.11±219.63
$t_{1/2z}$ [h]	3.39	3.99	3.07	3.48±0.47
T_{max} [h]	2.00	2.00	2.00	2.00±0.00
C_{max} [ug/L]	115.38	116.79	267.12	166.43±87.20

$V_{z/F}$ [L/kg]	162.18	159.92	86.19	136.10±43.24
$CL_{z/F}$ [L/h/kg]	33.16	27.75	19.45	26.79±6.91
	♀			
	**1**	**2**	**3**	**Mean±SD**
$AUC_{(0-t)}$ [ug/L·h]	885.16	1818.83	1232.02	1312.00±471.95
$AUC_{(0-\infty)}$ [ug/L·h]	897.84	1828.44	1234.21	1320.17±471.22
$t_{1/2z}$ [h]	3.63	3.00	2.43	3.02±0.60
T_{max} [h]	2.00	3.00	2.00	2.33±0.58
C_{max} [ug/L]	122.10	340.98	193.16	218.75±111.66
$V_{z/F}$ [L/kg]	116.76	47.28	56.79	73.61±37.67
$CL_{z/F}$ [L/h/kg]	22.28	10.94	16.21	16.47±5.67

Table 7. Effects of CK polymorphs on paw edema from rats induced by carrageenan.

	**0h**	**3h**	**4h**	**5h**	**6h**
Normal group	1.63±0.15	1.70±0.13<br>(4.87±13.01)	1.72±0.12<br>(5.61±9.53)	1.70±0.11<br>(4.44±6.89)	1.72±0.12<br>(5.56±8.86)
Model group	1.60±0.13	2.61±0.32<br>(64.27±23.44)##	2.58±0.21<br>(62.36±19.24)##	2.42±0.15<br>(951.76±14.42)##	2.22±0.10<br>(39.32±13.37)##
Indomethacin<br>1 mg/kg	1.60±0.13	2.35±0.15<br>(48.00±18.21)<br><25.32>	2.28±0.12<br>(43.75±15.39)<br><29.85>	2.08±0.15<br>(30.74±10.87)*<br><40.61>	1.97±0.12<br>(23.41±9.18)*<br><40.46>
From I<br>10 mg/kg	1.60±0.13	2.57±0.14<br>(61.02±11.75)<br><5.06>	2.42±0.19<br>(51.30±10.45)<br><17.74>	2.15±0.16<br>(34.69±9.89)*<br><32.99>	2.03±0.08<br>(27.53±8.13)<br><29.97>
From I<br>20 mg/kg	1.62±0.13	2.45±0.16<br>(52.64±19.00)<br><18.10>	2.30±0.11<br>(43.25±16.04)<br><30.65>	2.12±0.08<br>(31.96±15.31)*<br><38.25>	2.00±0.09<br>(24.52±13.39)<br><37.62>
From II<br>10 mg/kg	1.57±0.12	2.41±0.16<br>(55.25±18.20)<br><14.03>	2.30±0.14<br>(47.34±11.94)<br><24.09>	2.08±0.12<br>(33.73±13.72)<br><34.84>	1.98±0.08<br>(27.25±10.94)<br><30.70>
From II<br>20 mg/kg	1.65±0.14	2.40±0.15<br>(45.81±8.11)<br><28.72>	2.30±0.14<br>(39.71±6.48)*<br><36.33>	2.13±0.15<br>(29.62±8.06)**<br><42.77>	2.03±0.14<br>(23.59±8.59)*<br><40.00>
From III<br>10 mg/kg	1.60±0.11	2.50±0.06<br>(56.76±9.61)<br><11.69>	2.52±0.17<br>(58.19±18.49)<br><6.69>	2.27±0.14<br>(42.15±12.34)<br><18.56>	2.08±0.15<br>(30.81±14.08)<br><21.63>
From III<br>20 mg/kg	1.63±0.12	2.53±0.25<br>(55.29±13.62)<br><13.98>	2.42±0.13<br>(48.28±7.79)<br><22.59>	2.18±0.15<br>(34.20±12.28)*<br><33.94>	2.08±0.08<br>(27.94±7.14)<br><28.94>
From IV<br>10 mg/kg	1.60±0.05	2.62±0.13<br>(63.73±8.98)<br><9.41>	2.57±0.10<br>(60.32±7.19)<br><8.13>	2.26±0.05<br>(40.95±1.48)<br><18.24>	2.09±0.10<br>(30.95±10.46)<br><13.86>
From IV<br>20 mg/kg	1.62±0.05	2.64±0.16<br>(63.17±8.40)<br><10.20>	2.57±0.09<br>(58.65±3.69)<br><10.67>	2.26±0.13<br>(39.21±10.18)<br><21.72>	2.13±0.15<br>(31.27±12.96)<br><12.98>

(): paw edema; <>: edema inhibition; Values were expressed as mean±SD of 6 rats in group; *: p value < 0.05; **: p value < 0.01, *versus* the model group; #: p value < 0.05, ##: p value < 0.01, *versus* the normal group.
